# A Study on the Correlation of Various Factors in Patients With Severe Traumatic Brain Injuries

**DOI:** 10.7759/cureus.61877

**Published:** 2024-06-07

**Authors:** Nandkishor Sah, Abdur Raheem Khan, Himani Rathi

**Affiliations:** 1 Physiotherapy, Teerthanker Mahaveer University, Moradabad, IND; 2 Physiotherapy, Integral University, Lucknow, IND

**Keywords:** traumatic brain injury, glasgow coma scale, disability rating scale, recovery scale, unconsciousness

## Abstract

Introduction: Traumatic brain injury (TBI), ranging from minor impacts to severe cases, affects temporal and frontal brain areas, contributing to mortality and disability worldwide. The Glasgow Coma Scale (GCS) evaluates consciousness levels, aiding in prioritizing emergency care, while the Disability Rating Score (DRS) assesses overall function, particularly in severe cases, with greater sensitivity than GCS for clinical changes in TBI patients.

Objectives: To correlate various factors with each other in patients presented with severe TBIs.

Materials and methods: The retrospective study analyzed data from patients with severe TBIs admitted to the hospital from February 2023 to April 2024. Patients' demographic and clinical data, including GCS and DRS scores, were collected. Statistical analysis, including logistic regression, assessed mortality predictors.

Results: The study revealed significant correlations (p<0.05) between age and marital status (p=0.002) and surgery (p=0.003). Surgery also correlated significantly with the mechanism of injury (p<0.001). Furthermore, a negative correlation was found between GCS after 24 hours and change in GCS (p<0.001), while a positive correlation existed between DRS after 24 hours and DRS on the 14th day (p<0.001). These findings highlight the complex interplay between demographic factors, medical interventions, and clinical outcomes in TBI patients.

Conclusion: The study found that older individuals, particularly those involved in road traffic accidents, had poorer recovery outcomes and higher rates of surgery, with a strong correlation between changes in GCS and DRS scores.

## Introduction

Traumatic brain injury (TBI) can occur as a consequence of several incidents, ranging from minor head impacts to severe brain injuries. The temporal and frontal areas of the brain are the key areas involved in this phenomenon. Severe TBIs are those TBI cases that have attracted considerable attention due to their adverse cognitive effects in both civilian and military cohorts. Moderate to severe TBI is a prominent contributor to mortality and disability arising from physical trauma [1,2].

It is estimated that approximately 5.48 million individuals have severe TBI annually. According to the World Health Organisation (WHO), about 90% of fatalities resulting from injuries take place in low- and middle-income nations, which are home to 85% of the population. TBI is responsible for a significant proportion, ranging from one-third to one-half, of trauma-related fatalities. It stands as the leading cause of both mortality and disability on a global scale, surpassing all other trauma-related injuries [3]. The development of the Glasgow Coma Scale (GCS) aimed to classify persons with brain injuries based on their degree of consciousness. The primary purpose of developing this scale was to facilitate the evaluation and categorization of the dysfunction of the brain and its outcomes in a multicenter study including individuals with severe brain trauma [4]. The GCS is a commonly employed and acknowledged predictive measure for both traumatic and non-traumatic altered levels of consciousness. The assessment evaluates the optimal motor, vocal, and ocular reactions in individuals with TBI [5]. The GCS comprises three components: the best eye reply (E), the best motor response (M), and the best verbal answer (V). The reaction levels of the GCS are measured on a scale ranging from 1, indicating no response, to 4, indicating an eye-opening response, 5 indicating a verbal response, and 6 indicating a total response (also known as a motor response) [6,7]. Therefore, the comprehensive GCS spans from 3 to 15, where 3 represents the minimum value and 15 represents the maximum value. The combined total of the scores for each element constitutes the ultimate outcome [8,9]. The assessment of neurological function often includes evaluating responses in three main domains: eye responses, verbal responses, and motor responses. Eye responses can range from no opening of the eye to spontaneous opening, or opening in response to pain or sound stimuli. Verbal responses may involve no response, incomprehensible sounds, inappropriate words, confusion, or full orientation. Motor responses encompass a spectrum from no motor response to obeying commands, with intermediate stages such as pain-induced abnormal extension or flexion, pain withdrawal, and localization of pain. These assessments help clinicians gauge the level of consciousness and neurological integrity in patients, aiding in diagnosis and treatment planning [7-10].

The GCS is commonly employed for evaluating the responsiveness and early intervention of patients who have experienced a brain injury or other forms of acute brain injury. Patients who are more badly impacted are prioritized for emergency care decisions, such as preserving the triage and airway to determine patient transfer. Individuals with milder impairments are faced with the decision of whether to have neuroimaging, be admitted for observation, or get discharged from the hospital. Regular evaluation of the GCS is crucial for monitoring a patient's clinical advancement and guiding adjustments to treatment [11]. The Disability Rating Score (DRS) is a commonly employed assessment tool with eight items that are classified into four distinct groups. The scores go from 0 (indicating no disability) to 29 (indicating an absolutely vegetative condition). The scale in question is characterised by its conciseness, although it surpasses other concise measures in terms of comprehensiveness due to its inclusion of items that assess all three categories outlined by the WHO: disability, handicap, and impairment. The initial three components were developed by integrating elements from the GCS with minor adjustments. These elements are eye opening, motor response, and communication ability. The following items serve as indicators of impairment and hold significant importance in the observation and evaluation of behaviours in critically sick or emerging patients who exhibit severe limitations in their behavioural repertoire [12]. The highest attainable score on the DRS for a patient is 29, indicating a severe vegetative state. An individual without a disability would receive a score of zero. The scale is designed to precisely assess overall functional changes during the healing process. The simplicity of scoring and the conciseness of the scale are significant factors contributing to its widespread adoption. A preliminary investigation suggested that it exhibited greater sensitivity than the GCS in assessing clinical alterations in patients with severe TBI [13].

The study aims to evaluate correlations between various factors of patients presented with TBIs which would contribute clinically to understanding the prognosis of the patients.

## Materials and methods

Research design

The current retrospective study was conducted in our hospital, using the 130 patients’ data who visited our hospital with severe TBI from February 2023 to April 2024. GCS and DRS both were recorded upon the first visit and emergency interventions were provided. Each patient was given management based on the hospital’s protocol and the doctors’ advice. For this current study, the authors have obtained patients' data regarding age, gender, surgical history, type of injury and especially the GCS and DRS. These patients' data were obtained from the hospital and then arranged in the database. GCS and DRS were first measured after 24 hours of admission. The study considered GCS and DRS, again, after 14 days as a follow-up. The changes for each patient in the case of GCS and also DRS were determined. These parameters were evaluated with each other and a conclusion was drawn.

Inclusion criteria

The study included patients who were admitted to our hospital with severe TBI (with GCS 3 to 8) were only included. The patients between 10 and 50 years of age were considered. The study also included those patients whose assessments of GCS, DRS and other parameters were done after 24 hours of admission. Also, the patients who survived the hospitalisation and were discharged successfully were only included.

Exclusion criteria

Patients with “traumatic brain injuries” who were declared dead on arrival or for whom no medical records could be located for the registry were not included. Patients without enough data to assess TBI therapy and outcomes and those whose GCS were more than 8 were also excluded.

Statistical analysis

This data analysis used Statistical Package for the Social Sciences (IBM SPSS Statistics for Windows, IBM Corp., Version 27.0, Armonk, NY) to examine data distribution and create summary statistics for categorical and continuous variables. Frequencies and percentages were considered for categorical data, whereas mean values, standard deviations, and “interquartile ranges” were considered for constant variables such as age, gender, GCS, and DRS. Logistic regression was conducted between age and binary parameters. Bivariate correlation was conducted between most of the parameters while Pearson’s correlation was conducted between change in DRS and change in GCS.

## Results

A significant proportion of individuals fall within the 41 to 50 years age bracket, constituting approximately two-thirds of the sample population. This demographic trend suggests that individuals in this age range might be more susceptible to sustaining TBIs, whether due to occupational hazards, lifestyle factors, or other environmental influences. Additionally, the presence of a substantial number of participants in the 21 to 30 years age group indicates that young adults are also significantly affected by TBIs, which could be attributed to factors such as risky behaviours, sports-related injuries, or vehicular accidents prevalent in this demographic [4,5]. The age distribution showed that the majority of participants fell within the age range of 41 to 50 years, constituting 62.31% of the sample, followed by those aged 21 to 30 years (16.92%) and 31 to 40 years (14.62%).

Gender distribution shows a predominance of males in the occurrence of TBI, comprising nearly three-quarters of the total participants. This gender disproportionality is consistent with existing epidemiological data on TBIs, which often report higher incidence rates among males compared to females. The reasons behind this gender disparity could be multifactorial, involving differences in risk-taking behaviour, occupational exposure, anatomical variations, and sociocultural factors influencing help-seeking behaviours and healthcare utilization patterns [3,5]. In terms of gender, males were more prevalent, representing 74.62% of the sample compared to females, who constituted 25.38%. Table 1 shows the age and gender distribution of the study sample.

**Table 1:** Age and gender distribution of the study sample

Parameters	Number of Patients	%
Age range		
10 to 20 years	8	6.15
21 to 30 years	22	16.92
31 to 40 years	19	14.62
41 to 50 years	81	62.31
Sex distribution		
Male	97	74.62
Female	33	25.38

**Figure 1:**
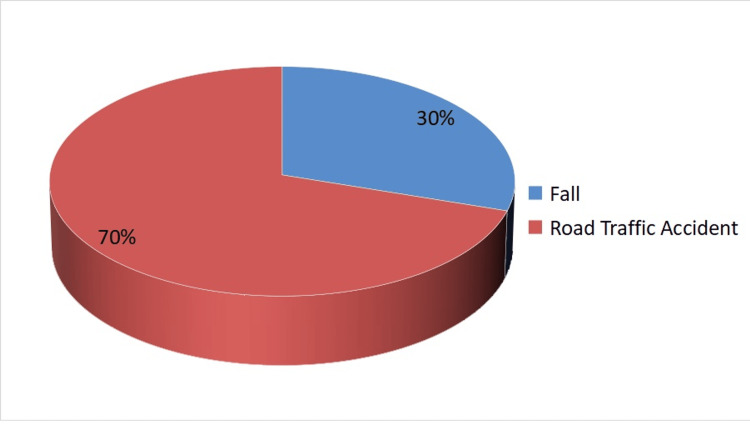
Mechanism of injury in patients of this study

Figure 1 shows the substantial prevalence of road traffic accidents as the primary mechanism of injury, accounting for the majority of cases (70% of cases). This may be associated with motor vehicle-related injuries which can be prevented by providing targeted interventions at the earliest possible and developing road safety measures, applying traffic regulations, and promoting awareness campaigns to mitigate the risk of vehicular collisions.

**Figure 2:**
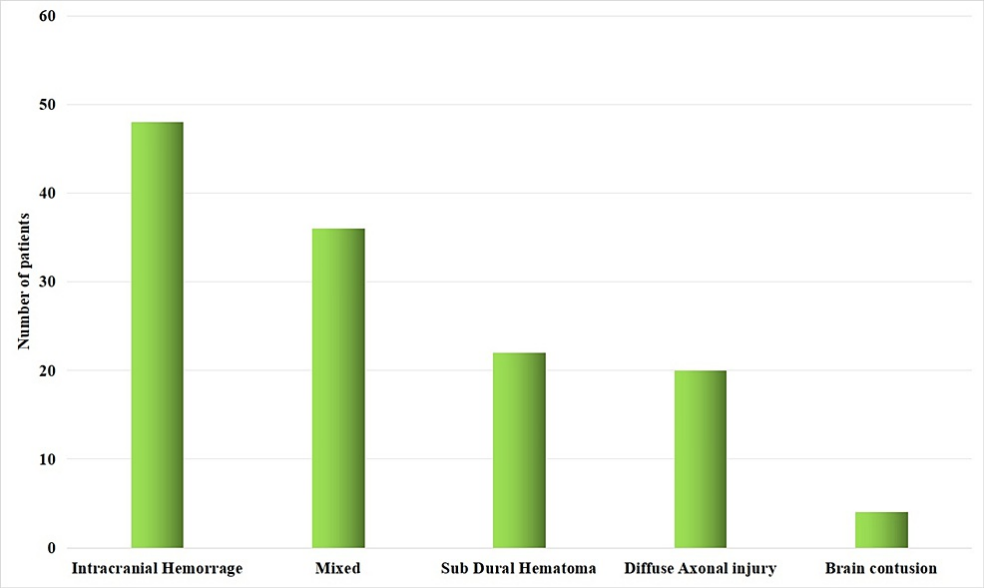
Types of brain injury in patients of this study

The study also showed that intracranial haemorrhage was the most common type of brain injury (36.92% cases), followed by mixed injuries (27.69%), subdural hematoma (16.92%), diffuse axonal injury (15.38%), and brain contusions (3.08%). Figure 2 shows the type of brain injury in this study.

Table 2 presents the mean scores of the GCS and DRS at two-time points: the first day and the follow-up on the 14th day. The mean GCS score on the first day was 5.95±1.15, indicating a relatively low level of consciousness among the patients at the initial assessment. However, by the 14th day, the mean GCS score had significantly improved to 9.89±1.59 which shows a significant recovery in neurological function over the two weeks. Again, the mean DRS score on the first day was 19.86±3.45, indicating a relatively high level of disability and impairment in functional outcomes immediately following the brain injury. However, by the 14th day, the mean DRS score had decreased to 14.92±3.03, signifying a significant reduction in disability and improvement in functional outcomes over the two weeks. This decrease in DRS scores reflects a positive trajectory in the patient's rehabilitation and recovery, with improvements in cognitive and physical functioning, independence, and social interactions observed over time.

**Table 2:** GCS and DRS with their mean scores on the first and 14th day GCS: Glasglow Coma Scale; DRS: Disability Rating Scale

Scoring and Time	Mean Score
GCS first day	5.95±1.15
GCS 14th day	9.89±1.59
DRS first day	19.86±3.45
DRS 14th day	14.92±3.03

Table 3 shows the relationships between various factors related to brain injury and recovery. Significant correlations (p<0.05) are observed between several variables. Notably, age shows significant positive correlations with surgery (p=0.003), indicating that older individuals are more likely to undergo surgery. Additionally, a significant positive correlation exists between surgery and the mechanism of injury (p<0.001), suggesting that the type of surgery is influenced by the mechanism of injury. Moreover, there is a significant negative correlation between GCS within 24 hours and change in GCS (p<0.001), implying that a lower initial GCS score is associated with a greater change in GCS over time. Similarly, a significant positive correlation is observed between DRS within 24 hours and DRS on the 14th day (p<0.001), indicating that a higher initial DRS score correlates with a higher score on the 14th day. These findings provide a correlation of various factors including demographic factors, medical interventions, and clinical outcomes in patients with brain injuries, pointing out the importance of considering multiple variables in treatment planning and prognostication.

**Table 3:** Correlation of factors between each other in this study GCS: Glasglow Coma Scale; DRS: Disability Rating Scale

	age	gender	marital_status	surgery	type_of_brain_injury	mechanism_of_injury	gcs within 24 hours	gcs on 14th day	drs within 24 hours	drs on 14th day
age	.	0.005	0.000	0.003	0.000	0.184	0.409	0.399	0.244	0.007
gender	0.005	.	0.110	0.351	0.007	0.760	0.346	0.581	0.836	0.309
marital_status	0.000	0.110	.	0.002	0.002	0.284	0.591	0.925	0.109	0.109
surgery	0.003	0.351	0.002	.	0.099	0.000	0.064	0.293	0.595	0.309
type_of_brain_injury	0.000	0.007	0.002	0.099	.	0.000	0.064	0.813	0.009	0.002
mechanism_of_injury	0.184	0.760	0.284	0.000	0.000	.	0.622	0.669	0.067	0.066
gcs within 24 hours	0.409	0.346	0.591	0.064	0.064	0.622	.	0.403	0.632	0.269
gcs on 14th day	0.399	0.581	0.925	0.293	0.813	0.669	0.403	.	0.790	0.290
drs after 24 hours	0.244	0.836	0.109	0.595	0.009	0.067	0.632	0.790	.	0.000
drs on 14th day	0.007	0.309	0.109	0.309	0.002	0.066	0.269	0.290	0.000	.

Table 4 shows the statistical significance measures regarding the relationship between changes in the GCS and changes in the DRS. The study conducted Pearson Chi-square analysis, showing a p-value of 0.000, indicating a highly significant relationship between the changes in GCS and DRS scores. Additionally, the linear-by-linear association test, which assesses the trend of association between the variables, shows a significant result with a value of 3.965 and a p-value of 0.046. This suggests that there is a linear relationship between changes in GCS and DRS.

**Table 4:** Significance between change in GCS and change in DRS GCS: Glasglow Coma Scale; DRS: Disability Rating Scale; df: degrees of freedom; *P<0.05 is considered as the level of significance.

Parameters	Values	df	P-value*
Pearson Chi-square	384.202	176	0.000
Likelihood ratio	131.560	176	0.995
Linear-by-linear association	3.965	1	0.046

## Discussion

A study by Rappaport et al. (1982) aimed to create a quantitative tool for evaluating the disability of patients with severe head injuries. This tool would enable the tracking of their rehabilitation progress from coma to various stages of awareness and functioning, ultimately leading to their reintegration into the community. The sensitivity of the DRS surpasses that of the GCS when it comes to identifying and quantifying clinical alterations in patients who have had significant head trauma. Additionally, this tool can be utilized to facilitate the identification of individuals who are most likely to derive benefits from intensive rehabilitation care in a hospital environment. It offers a concise and comprehensive overview of the status of a patient with a brain injury, which aids in comprehension and communication [14].

A prior investigation was undertaken to evaluate the correlation between the GCS at the time of admission and the length of survival in individuals diagnosed with TBI. Some patients with a GCS score above 8 may not survive due to the presence of specific comorbidities or the gradual buildup of intracranial blood. The study pointed out that the GCS may not serve as a precise predictor unless the patient's age and concurrent medical conditions are taken into consideration [15].

The majority of individuals who have experienced severe TBI are typically released from medical care before achieving full recovery. Several of these patients are not accessible for follow-up. A research investigation was undertaken to ascertain the potential correlation between the condition observed upon discharge from an acute care environment, as evaluated using the DRS, and the functional outcome observed throughout the follow-up period. All patients completed a DRS assessment on the day of their release. A comprehensive evaluation was conducted utilizing the Glasgow Outcome Scale Extended (GOSE) six months following the patient's release from the medical facility. The predictive accuracy of DRS for assessing GOSE at follow-up is satisfactory to excellent. If a more precise outcome assessment instrument becomes available, DRS at the time of discharge can serve as a substitute for GOSE at the follow-up [16].

The GCS is commonly employed as a means of evaluating the level of responsiveness and providing guidance for early intervention in cases of head injury or severe brain dysfunction. Patients with significant impairments may require magnetic resonance imaging (MRI), hospitalization for observation, or release, as decided by examinations using the GCS. A study was undertaken to assess the efficacy of GCS scores as an outcome measure which indicated that GCS can serve as a reliable predictor of outcomes for patients diagnosed with uncomplicated TBI [17].

A research investigation was undertaken to ascertain the correlation between the initial GCS score and the duration of mortality among adult patients admitted with TBI at the Princess Marina Hospital in Gaborone, Botswana. It substantiated a noteworthy correlation between the GCS and death. TBI primarily affected males. The external validity of these findings is compromised due to the limited sample size, necessitating the need for a larger multicenter investigation to establish validation [18].

A study examined the presence of correlations between variables during the initial phase of injury and measures of outcome in individuals with TBI. The combination of GCS scores, pupillary response, patient age, and broad outcome categories is the most precise method for predicting prognosis in head-injured patients. The prediction rates of the motor component of the GCS are comparable to those of the total GCS score. Furthermore, higher or lower GCS scores are associated with improved prediction accuracy [19].

Limitations of the study

The study's limitations include its retrospective design, reliance on data from a single hospital, limited time frame, and potential biases in data collection. These factors may restrict the generalizability of the findings and the ability to capture long-term outcomes in TBI management. Despite these constraints, the study offers valuable insights into the relationships between demographic factors, medical interventions, and clinical outcomes in severe TBI cases. Our study is limited to the cases that were discharged successfully and also found to have an improvement in 14 days of follow-up. This may impart selection bias to our current study.

## Conclusions

The study has concluded several clinically significant findings, notably a strong correlation between changes in GCS and DRS scores, a predominance of males, and intracranial haemorrhage emerging as the most common type of brain injury. The study has observed that more aged individuals recovered less and ultimately needed to operate mostly those individuals who had road traffic accidents. All these conclusions are clinically important in the context of rehabilitation and recovery in TBI patients, emphasizing the importance of ongoing monitoring and intervention to optimize outcomes.
